# fMRI Revealed Reduced Amygdala Activation after Nx4 in Mildly to Moderately Stressed Healthy Volunteers in a Randomized, Placebo-Controlled, Cross-Over Trial

**DOI:** 10.1038/s41598-020-60392-w

**Published:** 2020-03-02

**Authors:** Luisa Herrmann, Petya Vicheva, Vanessa Kasties, Lena V. Danyeli, Gregor R. Szycik, Dominik Denzel, Yan Fan, Johan Van der Meer, Johannes C. Vester, Herbert Eskoetter, Myron Schultz, Martin Walter

**Affiliations:** 1 Department of Psychiatry and Psychotherapy, University of Tübingen, Tübingen, 72076 Germany; 2 Department of Psychiatry and Psychotherapy, Jena University Hospital, Jena, 07743 Germany; 3 Clinical Affective Neuroimaging Laboratory (CANLAB), Magdeburg, 39120 Germany; 4 Medical Faculty, Otto von Guericke University of Magdeburg, Magdeburg, 39120 Germany; 5 Leibniz Institute for Neurobiology, Department of Behavioral Neurology, Magdeburg, 39118 Germany; 6 Department of Psychiatry, Social Psychiatry and Psychotherapy, Hannover Medical School, Hanover, 30625 Germany; 7 Department of Psychiatry, Charité-CBF, Berlin, 12203 Germany; 8 QIMR Berghofer Medical Research Institute, Brisbane, 4006 Australia; 9 idv Data Analysis and Study Planning, Krailling, 82152 Germany; 10 Medical Consultant, Cologne, 50737 Germany; 11 Heel GmbH, Baden-Baden, 76532 Germany

**Keywords:** Stress and resilience, Translational research

## Abstract

Social stress contributes to major societal health burdens, such as anxiety disorders and nervousness. Nx4 has been found to modulate stress responses. We investigated whether dampening of such responses is associated with neuronal correlates in brain regions involved in stress and anxiety. In a randomized, placebo-controlled, double-blind, cross-over trial, 39 healthy males took a single dose (three tablets) of either placebo or Nx4, 40 to 60 minutes before an fMRI scan session. We here report on drug effects on amygdala responses during a face-matching task, which was performed during a complex test battery further including resting-state brain connectivity and a social stress experiment. The first of the Primary Outcomes, defined in a hierarchical order, concerned reduced amygdala effects after intake of verum compared to placebo. We found a statistically significant reduction in differential activations in the left amygdala for the contrast negative faces versus forms during verum versus placebo condition. Our results indicate that effects of Nx4 can be monitored in the brain. Previously noted effects on stress responses may thus be modulated by affective brain regions including the amygdala.

## Introduction

More than half of the population in Germany suffered from stress in the past five years, and almost 25% suffered frequently^1^. But what is stress? Hans Selye influenced the definition of the term “stress”, describing it as “the non-specific response of the body to any demand for change”^2^. The reaction of the body to any unexpected positive or negative stimuli from external and internal cues for survival was defined to be stress. Nowadays, the term “stress” usually refers to a state of tension. Depending on the cognitive appraisal of the physiological outcome and the stress-inducing stimulus, a highly recurrent and long-lasting tension state may lead to a maladaptive reaction to the stressor and an increase in physio- and psychopathology^3–5^. Chronic stress is assumed to be a major risk factor for several physical and psychological disorders leading to increased heart rate and blood pressure, raised cholesterol, headache, restlessness, inability to concentrate, fatigue, state of anxiety, and feeling of depression^6^.

The stress response is mainly driven by the autonomic nervous system (ANS) and the hypothalamic-pituitary-adrenal (HPA) axis^7,8^. For this autonomic and neuroendocrine stress response, the hypothalamus and brainstem are the most important brain regions. However, higher cognitive regions involved in memory consolidation, anxiety, and decision making are also targeted by stress and stress hormones^7^. During situations of heightened arousal, such as stress or emotional experiences, noradrenaline is released, having an effect on the amygdala. Acute stress sensitizes the amygdala but lowers the specificity of its response; therefore, discrimination between stimuli becomes more difficult. However, in harmful situations, the recognition of any potential risk can be advantageous^9^. The basolateral complex of the amygdala (BLA) interacts with other brain regions, such as the insula, anterior cingulate cortex (ACC), and the prefrontal cortex to mediate stress and emotional effects on memory consolidation. Since memory consolidation of highly emotional experiences is more efficient, extremely disturbing situations can lead to traumatic and fearful memories, potentially resulting in symptoms of anxiety disorders^10^. Exposure to acute and chronic stress translates into morphological changes, namely an increased number of spines in the BLA and medial amygdala, thereby enhancing synaptic connectivity. This might be the neuronal basis for a raise in anxiety^10^. Hölzel *et al*. found a positive correlation of Perceived Stress Scale (PSS) scores and grey matter density in the BLA after a stress-reducing intervention^11^. The greater the decrease of the perceived stress, the greater was the decrease of grey matter. This finding indicated that a reduction of the stress level by reappraisal of the outcome of the emotional stress response may even result in morphological changes in the amygdala. Previous studies showed an increased activation of the amygdala in response to fearful facial expressions, even when the faces were masked^12,13^. Especially the left amygdala was shown to be stronger activated during angry or fearful face processing in contrast to neutral or happy faces^14–16^. The left amygdala was also more discriminatory than the right one in distinguishing between fearful eyes and gaze shifts^12^. This suggests lateralization effects for the amygdala in the context of stress and emotion processing. Baas *et al*. found early metanalytical support in more than 50 studies for the notion of a more consistent or more frequent activation of the left amygdala than the right amygdala in emotion processing independent of stimulus type or task instruction^17^. Furthermore, studies examining the effect of positive and negative faces in participants with stress disorders revealed that patients suffering from PTSD show heightened amygdala responses to fearful faces in contrast to happy faces^18,19^. The activation in the left amygdala was also increased by trauma-relevant negative words in contrast to neutral words in healthy subjects^20^. This effect was increased in patients with PTSD.

In order to reduce the consequences of a long-lasting tension state, such as nervousness, restlessness, and insomnia, people often take stress-relieving drugs like the commonly used valerian. Neurexan^®^ (Nx4) is an alternative sleep-inducing drug, which has become available in the last couple of years. Nx4 is a medicinal product, consisting of three herbal extracts (Avena sativa, Coffea arabica, Passiflora incarnata) and one mineral salt (Zincum isovalerianicum) in low but measurable concentrations. When comparing the sleep-inducing effects of Nx4, the natural, multicomponent medicine investigated in our study, and valerian, Waldschütz and Klein found comparable effectiveness for both drugs^21^. Nx4 reduced sleep latency and significantly increased the duration of sleep in a sample of 156 participants receiving Nx4. In addition, significantly more of these participants reported a lack of daytime fatigue under Nx4 (49%) compared to participants receiving valerian (32%, p < 0.05). An observational study conducted in 49 German general practices also showed a significantly greater reduction of nervousness/restlessness in participants taking Nx4 (reduction of 11.5 ± 7.3 score units) for two weeks in customized doses than participants taking valerian (reduction of 9.0 ± 6.6 score units) for two weeks (p < 0.001)^22^. Nx4 was also shown to significantly (p < 0.001) change EEG signatures in rats that received a single oral dose of Nx4 in comparison to a vehicle control^23^. Dimpfel *et al*. found altered delta- and theta-waves in the frontal cortex and reticular formation, suggesting a calming effect of Nx4^23^. The efficacy of Nx4 on psychological and neuroendocrine responses under acute stress was recently investigated by Doering *et al*.^24^. They found a significantly diminished neuroendocrine response to an acute stressor (i.e., the Trier Social Stress Test [TSST]) after intake of Nx4 compared to placebo. In particular, Nx4 reduced salivary cortisol (time x group interaction, F[1,57] = 4.69, p = 0.035) and plasma adrenaline levels (time x group interaction, F[1,50] = 5.19, p = 0.027) after stress exposure, but not the subjective feeling of stress or heart rate and blood pressure under acute stress. The demonstration of placebo-controlled effects, as reported by Doering *et al*.^24^, are supportive for further investigations including those for a mode of action. Lacking any evidence for brain regional effects in humans, the mode of action of Nx4, however, still remains largely unclear. Considering that Nx4 has been shown to be effective against symptoms of stress and because the amygdala plays a key role in processing emotions, such as fear or anxiety, we hypothesized that Nx4 may reduce amygdala activation and connectivity in response to negative stimuli. This study was thus designed to test if Nx4 is related to any measurable brain responses, when compared to placebo and, testing specific regional hypotheses, to give a first insight into a potential mode of action of Nx4 in humans.

To induce stress inside an MR scanner, Streit *et al*. developed the ScanSTRESS analogue to the TSST^25^. The psychosocial stress induction during MR measurements revealed activation of the ventral striatum, thalamus, insula, hippocampus, and the amygdala as well as deactivation of the rostral ACC^25^. Even one hour after the TSST, the functional connectivity of the amygdala with the posterior cingulate cortex and the precuneus, which play a role in emotional processing, was increased^26^.

In the current study, healthy male participants, who, however, already experienced mild to moderate chronic stress, were exposed to acute stress to examine the efficacy of Nx4 in reducing amygdala activation. In order to control for any non-pharmacological effect, Nx4 was tested against placebo in a double-blind manner. The benefits of a cross-over design compared to a parallel group design include a diminished influence of confounding covariates (because each participant serves as their own control), an increase in statistical power, and a reduction in sample size^27^. We focused on emotional processing and social stress; therefore, the amygdala was our main region of interest (ROI). In order to specifically activate the amygdala and test the effect of Nx4 on the emotional brain response reported in this manuscript, we performed an emotional face-matching task (Hariri task^28^) with a comparison of forms condition as control. Based on previously observed stress-reducing effects of Nx4, we hypothesized a reduced amygdala response after treatment with Nx4 and tested if this reduction correlated with stress- induced subjective nervousness measured by a visual analogue scale (VAS).

## Results

The study was performed at the Medical Faculty of the Otto-von-Guericke University of Magdeburg, Germany, from August 18^th^, 2015 to December 3^rd^, 2015. MRI and EEG experiments took place at the local imaging center of the university. Participants fulfilling the inclusion criteria were recruited from July 2015 to November 2015. All screenings and measurements took place from August 2015 to December 2015. In this report, we focus on the first primary outcome, concerning Hariri task, while other findings from this extensive study will be reported elsewhere.

**Figure 1:**
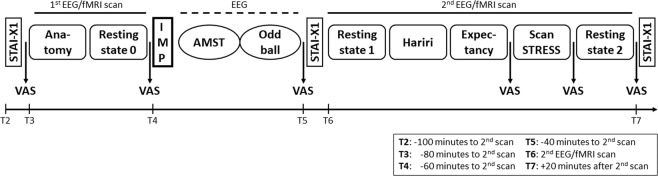
Design of the cross-over MRI sessions. MRI acquisition began with an anatomical scan followed by a baseline resting-state measurement. After intake of the IMP, the two EEG paradigms (AMST and Oddball) were performed. The second MRI scan included three task measurements, starting with the Hariri paradigm, and two resting-state measurements before and after the tasks, respectively. Anxiety was measured by STAI-X1 and was assessed before the first and second scan as well as at the end of measurements. Nervousness measured by VAS was gathered six times during the day; data before and after stress induction were of special interest. MRI = Magnetic Resonance Imaging; IMP = Investigational Medicinal Product; EEG = Electroencephalography; AMST = Attention Modulation by Salience Task; STAI = State-Trait Anxiety Inventory; VAS = Visual Analogue Scale; T = time point.

Each of the two days (one for placebo and one for verum intake) included five tasks, three of them inside the scanner and two with EEG only, three resting state sessions inside the scanner and psychometric assessments (Fig. 1). Participants were aged 31 to 59 and reported mild to moderate stress scores, particularly with PSS^29^ score ranging from 10 to 24 and Trier Inventory of Chronic Stress (TICS)^30^ score from 9 to 30. Groups of different order of IMP did not differ in these variables. Mean age and stress scores are shown in Table 1. All 39 participants who finished the study (20 participants receiving verum first and 19 participants receiving placebo first) for whom full data were available were included in the analysis of the Hariri task (Primary Outcome 1). One participant did not complete the study due to an incidental MR finding. No participant had to be excluded due to motion. We did not observe any adverse events during the entire study.

**Table 1:** Mean age and stress scores (±SD) of the two study groups receiving first Nx4 and placebo, respectively.

	Verum first	Placebo first
n	20	19
Age	43.2 ± 10.00	43.7 ± 9.61
PSS	14.1 ± 3.54	15.5 ± 4.06
TICS-SSCS	15.5 ± 5.81	15.5 ± 5.85

### Psychometric measures

Nervousness measured by VAS increased after inducing stress via ScanSTRESS task compared to the assessment immediately before the task, but this subjective rating of nervousness did not differ significantly between verum and placebo condition (t(38) = 1.75, p = 0.089).

### Primary Outcome 1 (Hariri Task fMRI)

#### Reproduction

**Figure 2:**
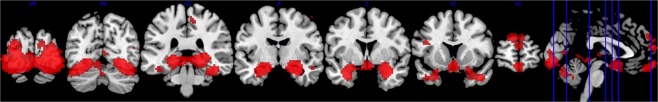
Activation pattern in the contrast negative faces versus forms in placebo only on whole-brain level. Voxel threshold p < 0.05 (peak-level FWE-corrected).

Firstly, we validated the task effect by comparing our findings with previously reported findings. When comparing negative faces and forms during the placebo condition on a whole-brain level, we found significant bilateral activations in the fusiform gyri, amygdalae and prefrontal cortex as well as a significant, unilateral activation in the right thalamus. These findings have also been reported by Hariri *et al*.^28^, who proposed the emotional paradigm used here. Additionally, there were significant activations in visual cortex and cerebellum. All activations are illustrated in Figure 2.

#### Amygdala response under Nx4

**Figure 3:**
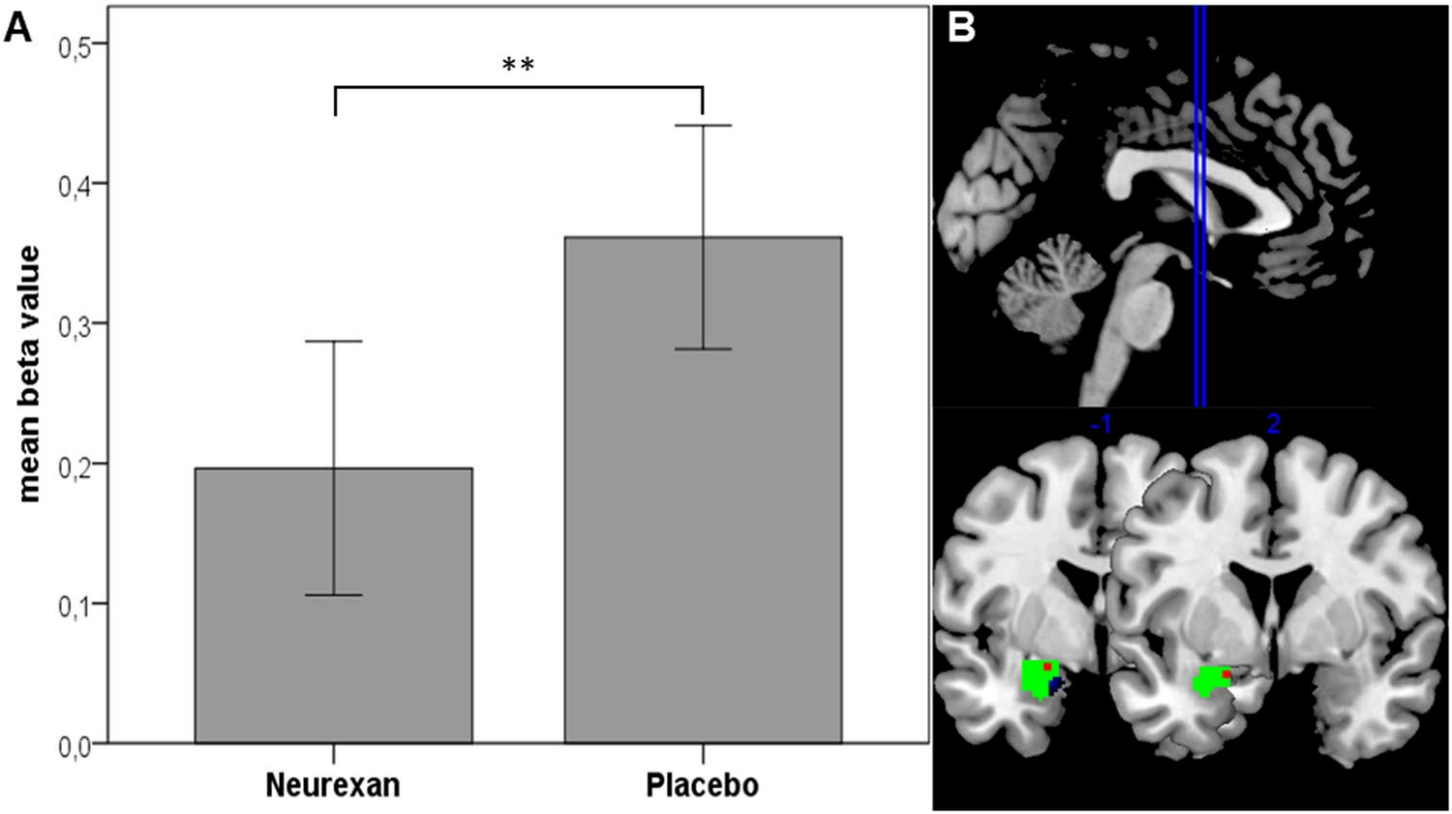
Differential activation in the left amygdala is significantly different in the contrast negative faces versus forms in verum compared to placebo conditions (paired t-test: p = 0.004, t = -3.101; GLM corrected for treatment order: p = 0.003, F = 9.764). The bar diagram of mean beta values (±SE) showed that the differential amygdala activation (negative faces - forms) is reduced in the Nx4 condition. A follow-up voxel-wise analysis revealed amygdala-wide (B, ROI in green) centering of strongest effects for this contrast in two voxels (p = 0.035, peak-level FWE-corrected after SVC; MNI coordinates -18 2 -16 and -21 -1 -13; B; significant voxels in red) at a cortico-median location of the amygdala (B; centrocortical amygdala cluster based on Bach *et al*.^56^ in blue).

Secondly, beta values were extracted from the ROI, the left amygdala as defined by Automated Anatomical Labelling Atlas (AAL), and paired t-test analysis revealed a significant drug effect. Comparison of mean beta values (0.196 for Nx4 versus 0.361 for placebo) indicated significantly reduced differential activations for the contrast negative faces versus forms under Nx4 condition when compared to placebo within the left amygdala ROI (p = 0.004, t = -3.101, Fig. 3A). Additional inclusion of the individual treatment order of drug and placebo intake using a general linear model (GLM) for repeated measures revealed equally significant outcome (p = 0.003, F = 9.764).

#### Location of maxima

Thirdly, the activation in the left amygdala masked for the ROI is depicted in Fig. 3B. Analysis of the locus of drug modulated differential activation showed a cluster of two adjacent voxels, peak-level significant after small volume correction (SVC) for the search volume (p = 0.035, peak-level family wise error [FWE]-corrected, T = 3.12, Montreal Neurological Institute [MNI] coordinates -18 2 -16 and -21 -1 -13) located in a left cortico-median portion of the amygdala.

### Secondary outcome (correlation with psychometric measures)

**Figure 4:**
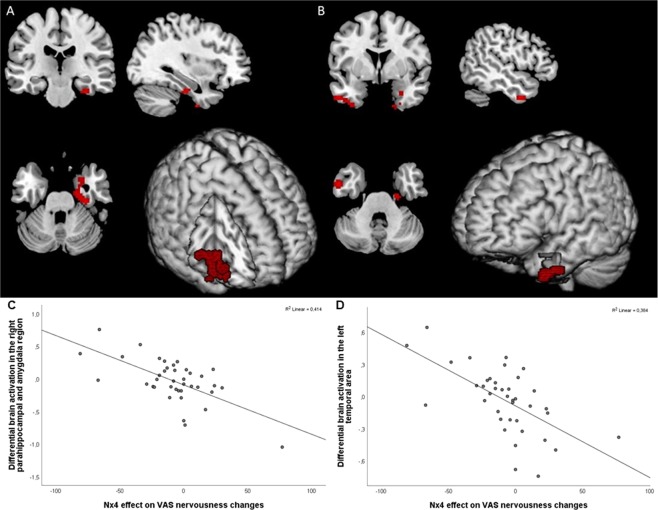
Correlation of Nx4 efficacy on the Hariri task with the Nx4 efficacy on VAS nervousness before versus after stress showed brain activations in a right parahippocampal-amygdala region (cluster-level FWE-corrected p = 0.011; (**A**)) as well as trend-wise in a left temporal area (cluster-level FWE-corrected p = 0.065; (**B**)). Scatterplots show the negative correlation between Nx4 efficacy on VAS nervousness changes and differential brain activation in the right parahippocampal-amygdala region (**C**) as well as in the left temporal area (**D**).

We then correlated the Hariri second level contrast negative faces > forms with the subjective reports of nervousness measured by VAS before versus after stress in the Nx4 condition compared to placebo. Differential brain activation in a right parahippocampal-amygdala region (cluster-level FWE-corrected p = 0.011, k = 125, MNI coordinates 33 -7 -46) and, on trend level, in a left temporal area (cluster-level FWE-corrected p = 0.065, k = 78, MNI coordinates -51 2 -34) correlated negatively with the Nx4 efficacy on VAS nervousness before versus after stress (Fig. 4A,B). A dampened increase in nervousness after inducing acute stress in the Nx4 compared to placebo condition is associated with reduced activity in parahippocampal and temporal areas (Fig. 4C,D).

### Exploratory results

#### Drug effect on whole-brain level

**Figure 5:**
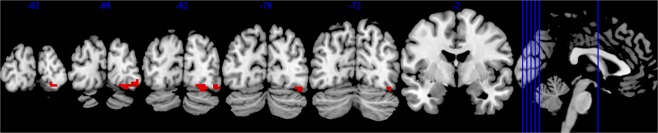
Cluster of trend-level difference in the contrast negative faces versus forms in the placebo versus Nx4 condition on whole-brain level. Voxel threshold p < 0.001 (cluster-level FWE-corrected p = 0.086, k = 67, MNI coordinates 27 -85 -16, initial voxel height threshold p < 0.001).

We explored the effect of Nx4 during negative faces on whole-brain level to identify potential other regions affected by Nx4. When comparing the contrast of negative faces > forms in the placebo versus Nx4 condition, we found a cluster of trend-level difference in the right fusiform gyrus (cluster-level FWE-corrected p = 0.086, k = 67, MNI 27 -85 -16), but there was no whole-brain significant cluster in the amygdala (Fig. 5).

#### Drug effect within the left amygdala

When we examined the effect of Nx4 versus placebo in the second level within the first level contrasts all faces > forms and positive faces > forms, we did not find any significant differential activation in the left amygdala after correcting for multiple comparisons across all voxels within the ROI (SVC). Further, no voxels were found which surpassed even a more liberal uncorrected height threshold of p < 0.005.

## Discussion

Our primary hypothesis was that Nx4 reduces amygdala activation in response to negative stimuli. This was confirmed by statistically significant reductions of contrast estimates of negative faces - forms in the left amygdala in the verum versus placebo condition. The task effect in our study was validated by comparing our findings with the previously published results of Hariri *et al*.^28^, which were in accordance. The local activation changes in an amygdala-parahippocampal cluster during an emotional face-matching task in the Nx4 versus placebo condition were negatively correlated with the effect of Nx4 on subjective rating of nervousness before versus after stress when compared to placebo conditions, which supports an interpretation of drug effects in the Hariri task along with reduced stress related brain activity.

The amygdala plays a key role in efficient processing of emotions^10^ and is sensitized by acute stress^9^, which in turn leads to an increase in anxiety. Therefore, we expected to find differences in amygdala activation between Nx4 and placebo. Such differential activation was found in the cortico-medial portion of the amygdala, which is known to directly influence the ANS and modulate the release of stress hormones via afferents to the hypothalamus. In this way, the cortico-medial amygdala influences behavioral responses to stress^31^ and causes a perceived feeling of stress.

The favorable effect of Nx4 on stress markers and its clinical effectiveness in insomnia and in nervousness/restlessness have previously been reported^21,22,24^. However, no reduction in the subjective stress ratings induced by the drug was reported. It is possible that the interindividual variability in our sample might have statistically overshadowed the inter-group differences, similarly to what was shown by Doering *et al*.^24^. Furthermore, Doering *et al*. found a general effect of gender, with men reporting less nervousness and tension compared to women^24^. Arguably, only including male participants might have influenced the subjective ratings and might explain why there was no difference in the reported stress levels between the groups. Amygdala activation changes have been reported for anxiolytic drugs^32,33^. In an fMRI study, Cornelius *et al*. found decreased amygdala reactivity during an emotional face-matching task when the level of cannabis use was increased^34^. In the present study, we have confirmed that Nx4 reduces amygdala activation in fMRI as well.

Critically, one has to say that exploratory whole-brain findings of activation cluster examining the drug effect during the processing of negative faces did not reveal significant results for any additional amygdala cluster bilaterally when correcting for whole-brain analyses. Clusters of differential activation in other regions (e.g., parahippocampal regions) were also not significant when correcting for multiple comparisons, despite their known interconnectivity with the amygdala^35,36^. However, subjective nervousness ratings in the Nx4 treatment group did correlate with reduced parahippocampal activation differences during Nx4 when compared to placebo. These regions are thought to be part of the ventral stream of visual processing, which is commonly thought to proceed along two distinct pathways, a dorsal “where-pathway” for spatial vision, projecting into the parietal cortex, and a ventral “what-pathway” (also called ventral stream) for object recognition, projecting into the temporal cortex^37^. The parahippocampal finding suggests that Nx4 efficacy on nervousness has a neural correlate in the ventral stream activation. Notably, this region is influenced by activations in the amygdala, where a primary effect of Nx4 was found. This significantly negative correlation supports our hypothesis that Nx4 reduces markers of stress, as a reduced increase in subjective nervousness ratings is indicated by high values of Nx4 efficacy (strong effect of Nx4) when comparing pre- and post-stress and a reduced differential activation of the ventral stream is indicated by low values of Nx4 efficacy (strong effect of Nx4). This correlation indicates that regions along the visual stream could be targets of Nx4 biological action in the brain, at least regarding the subjective stress level. In a review, Kravitz *et al*. showed a much more complex connectivity pattern of the ventral stream and summarized evidence about its role in learning, memory, and emotional processing^37^. They also reported an occipitotemporal-amygdaloid pathway involved in the processing of emotionally salient stimuli. Another study also provides a link between the ventral stream and the appraisal of emotional behavior and the production of an affective state^38^. Therefore, Nx4 could have a modulating effect in the early stages of neural processing of stressful stimuli by reducing the neural activity in the regions of the ventral pathway and, consequently, lowering the perceived nervousness.

One limitation of the present study is that our sample was specific in that we excluded participants with very low and very high stress scores; therefore, our results need to be tested for participants with greater everyday burden or even stress-induced diseases. Further, the Nx4 effect on the amygdala during the negative faces condition did not show a significant result on whole-brain level. Within the amygdala a confidential confirmation of the peak location in one of the amygdala subregions needs to be taken with great caution due to the suboptimal resolution of the data, the small size of the amygdala and general imprecision of the vascular BOLD signal downstream of its neuronal origin and inhomogeneous signal to noise ratio within the amygdala potentially biasing localized observations^39^. Moreover, the subjective stress ratings did not show a main drug effect itself, which may be related to the variability between participants; hence, the significant correlation with Nx4 efficacy during the Hariri task has to be interpreted with caution.

Our findings support the hypothesis that Nx4 modulates emotional face processing in a region involved in face recognition and social stress and anxiety. The treatment effect depends on interindividual variability, with participants showing the strongest brain effects in the amygdala also showing the strongest effect on nervousness.

In conclusion, Nx4 reduces the brain response to negative emotional stimuli in a brain region known to be important in stress and anxiety. Our results encourage a randomized, placebo-controlled trial in patients suffering from chronic stress with a larger sample size and patient-reported outcomes.

## Methods

This study is registered at ClinicalTrials.gov, number NCT02602275 (date of registration: October 28^th^, 2015). It was approved by the ethics committee of the University of Magdeburg as well as the Competent Authority (Federal Institute for Drugs and Medical Devices) and conducted in compliance with ethical principles of the 1996 Declaration of Helsinki (Somerset West, Republic of South Africa), principles of the Good Clinical Practice (GCP) provided in the International Conference on Harmonisation (ICH) Harmonised Tripartite Guidelines for GCP 1996, and all applicable national laws and regulations. Each participant signed a written informed consent document prior to any study procedure. To ensure study rigor and transparency the study protocol was agreed upon in an international scientific advisory board meeting by external independent experts not otherwise involved in the study. After the completion of the study the results were challenged and agreed upon in an additional international scientific advisory board meeting by independent experts not otherwise involved in the study. The full data of the study are currently not publicly available due to ongoing additional analyses, however, individual summary data concerning this manuscript may be available on reasonable request from the authors.

### Trial design

The study was a randomized, placebo-controlled, double-blind, two-period cross-over trial with an explorative design with confirmatory principles. For every participant, the study consisted of three visits. Medical and psychological symptoms were screened on Day 0. Each participant completed psychometric tests about personality traits, life experiences and sensitivities between 3 to 7 days before randomization (Day 1). At bedtime the evening before and in the morning of Day 1 after awakening, the participant collected saliva to determine the cortisol awakening response (CAR). Randomization and study procedures including MRI and electroencephalography (EEG) data acquisition were performed on Day 1. The cross-over MRI session (Day 2) took place 7 to 35 days (washout period) after Day 1. Participants who received verum on Day 1 got placebo on Day 2 and vice versa. Apart from that, study procedures on Day 1 and Day 2 were identical. On Day 1, MRI measurements with simultaneous EEG data acquisition started with a first scan of anatomy and 12-minute baseline resting-state scan before intake of the Investigational Medicinal Product (IMP; Fig. 1). The participant took a single dose (three pills) of Nx4 or placebo 40 to 60 minutes prior to the second scan. Between the first and the second scan, the participant performed two computer tests, the Attention Modulation by Salience Task (AMST) and an auditory oddball task, while EEG data were acquired. The second MRI scan was acquired with simultaneous EEG data collection and comprised three tasks: first, the Hariri task; second, the Expectancy task; and, third, the ScanSTRESS task; as well as a pre-task resting-state and a post-task resting-state scan of 12 minutes each. Concurrent EEG was necessary to ascertain awake state during resting-state runs given the literature on sleep incidents^40^ and visual inspection of EPI data was performed to exclude potential signal losses due to the EEG net (see Supplementary Figure S1). To investigate the physiological stress response further, saliva samples for cortisol measurements were collected directly before and after stress induction and again 20 minutes and 40 minutes after stress induction. The subjective feeling of anxiety was assessed by the State-Trait Anxiety Inventory (STAI-X1) at baseline, before the second MRI scan, and at the end of Day 1. Furthermore, subjective feeling of nervousness was assessed by a continuous VAS^41^ reaching from 0 to 100 before and after the first MRI scan, before and after the second MRI scan as well as before and after ScanSTRESS. The participants were asked to rate their subjective impression of nervousness on a 10 cm bipolar visual scale ranging from 0 = “not at all” to 100 = “highly”.

### Task design

**Figure 6:**
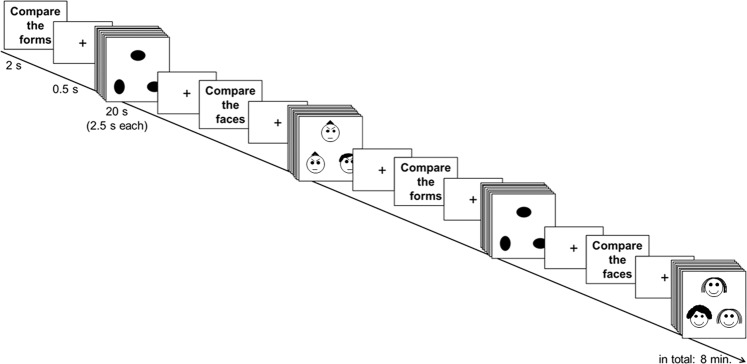
The Hariri task is a face-matching task with a form condition as control. Each block begins with a cue announcing the current condition (face or form) for 2 seconds, followed by a fixation cross shown for 0.5 seconds. Eight trials of face-matching or form-matching, respectively, were presented during each block, and each test was displayed for 2.5 seconds, adding up in blocks of 20 seconds. In total, the eight-minute task consisted of five blocks with happy faces, five blocks with negative (afraid and angry) faces and ten blocks with forms. Pictures of head-on faces were taken from the Karolinska Directed Emotional Faces (KDEF) set, which has been used in several experiments before^42,57–60^.

The Hariri task, on which we focus in this manuscript, is an emotional face-matching task. This task induced an amygdala response and, thus, allowed us to investigate the effect of Nx4 on emotional response. In a block design, facial expressions showing negative (fear and anger) and positive (happiness) emotions as well as forms were presented. A schematic illustration of the block design is shown in Fig. 6. In each trial, a reference (i.e., either an emotional face or an oval vertical or horizontal form) was presented on the top of the computer screen. Two samples (faces or forms, respectively) were presented at the bottom of the screen. Eight consecutive trials were presented during each block, and each trial was displayed for 2.5 seconds, with an interstimulus interval jittered between 0.5 - 1 second. Blocks were presented in alternating order and were separated by a fixation cross (duration = 0.5 seconds) and a “cue” (duration = 2 seconds) indicating the following block. Participants were instructed to match the sample with the same emotional expression or form as shown in the reference by pressing the left or the right key on a button box. Color pictures of facial expressions were selected from the Karolinska Directed Emotional Faces (KDEF) set^42^. Five blocks of positive emotions, five blocks of negative emotions, and ten blocks of forms were presented during the eight-minute MRI data acquisition. The exact KDEF stimuli will be provided by request.

The Hariri task was followed by two other tasks inside the scanner that will be reported in separate manuscripts. The emotional Expectancy task used visual cues and pictures inducing different emotions^43^. A total of 60 pictures divided in positive, negative, and neutral emotion were selected from the International Affective Picture System (IAPS). Half of the pictures from each category were preceded by a visual cue (expectancy period) and half were presented instantly after fixation period with no visual cues. Visual cues depicted by an arrow pointing up indicated a positive picture (followed 10 times by a picture and 5 times by fixation periods), an arrow pointing horizontally indicated a neutral picture and an arrow pointing down indicated a negative picture (followed 10 times by a picture and 5 times by fixation periods). The purpose of the cue-picture-mismatch events was to bring new stimuli to the participants therefore increase their attention to pictures. The same number of pictures followed directly after fixation. Each picture presentation was followed by a fixation period depicted by a small cross in the middle of the screen.

During the ScanSTRESS acute psychosocial stress was induced with an adapted version of the Montreal Imaging Stress Task^44,45^ previously published as ScanSTRESS by Streit *et al*.^25^. The task is an fMRI-compatible adaptation of the Trier Social Stress Task (TSST^46^;) containing arithmetic tasks and mental rotations. For both types of task control blocks without any social evaluative feedback, time pressure, or difficult questions and stress blocks with feedback about the correctness and speed of the answers as well as more demanding questions alternated. During the whole experiment two panel members observed the performance of the participants, who were continuously exposed to a video feed of the reactions of the panel. While the panel members were passive during the control blocks, they reacted disapprovingly to the participants’ performance during the stress condition. In this report, we only focus on ScanSTRESS induced changes in nervousness rating as behavioral correlates of Nx4 effects during the Hariri task. Extensive analyses on the neural effects during the ScanSTRESS task will be reported elsewhere.

### Resting state

One run of rest took 12 minutes and subjects were asked to keep their eyes closed and not engage in any specific tasks while trying not to fall asleep^47^. Throughout the study, three resting state runs were performed, one before IMP intake, one after IMP and before fMRI and one at the end the of the fMRI session. Thus, the Hariri task was performed 12 minutes after the subjects entered the scanner for the second time (after taking the IMP outside the scanner).

### Participants

In this study, 40 healthy, mildly to moderately stressed male participants aged 31 to 59 years were enrolled. During baseline MR measurements, one subject was excluded due to an incidental finding thus 39 subjects were treated in this study. Healthy for the purpose of screening means without any lifetime episode of any mental disorder and abuse of any substance in respect of a Structured Clinical Interview for Diagnostic and Statistical Manual of Mental Disorder 4^th^ edition (DSM-IV) Axis I (SCID), assessment of medication use, medical examination and history as well as blood count. Stress level was assessed during a screening visit by PSS and the screening scale for chronic stress (TICS-SSCS). Participants with high chronic stress (TICS-SSCS score > 36) and low chronic stress (PSS score ≤ 9, TICS-SSCS score < 9) were not included in the study to (1) ensure that participants are in principle susceptible to stress and (2) avoid a ceiling effect of stress sensitivity. All participants needed to be nonsmokers for at least three months, MRI compatible, fluent in German, and able to understand the explanations and instructions given during the study; in addition, they had to be willing to adhere to all specified prohibitions and restrictions.

We excluded participants with the following attributes: a current or past history of psychotic features or a diagnosis of any psychiatric disorder defined in the DSM-IV Axis I; use of any psychotropic medication or intake of prescription drugs for sleeping disorders or nervousness within one month prior to the screening visit; intake of over-the-counter (OTC) medication for the treatment of sleeping disorders or nervousness within the last week prior to the screening visit; blood pressure (BP) ≥ 160/100 mmHg at the screening visit and at randomization; treated hypertension; known allergies and/or hypersensitivity to ingredients of Nx4 or placebo, e.g. known lactose intolerance; use of any psychological stress-management intervention within the last four weeks prior to the screening visit; a history of substance, drug, nicotine, or alcohol abuse within the preceding three months prior to the screening visit; intake of alcohol, drugs, or nicotine within the last 24 hours before the screening visit and before randomization; a body mass index (BMI) > 30 kg/m^2^; regularly night shifts; serious, unstable illnesses including hepatic, renal, gastroenterological, respiratory, cardiovascular, endocrinological, neurological, immunological or hematological disease; any somatic disease or other condition the investigators or their duly assigned representatives believe could either affect the ability of the individual to complete the study or the interpretation of the study results; medical illness that may have influenced brain morphology and/or physiology; a history of one or more seizures without a clear and resolved etiology, claustrophobia, tinnitus, clinically significant acute illness within seven days prior to randomization; presence of metallic (ferromagnetic) implants, tattoos, or piercings; and participants that have received an experimental drug or used an experimental medical device within the last 30 days before study inclusion or whose ability to speak for themselves lacks or can be doubted.

**Figure 7:**
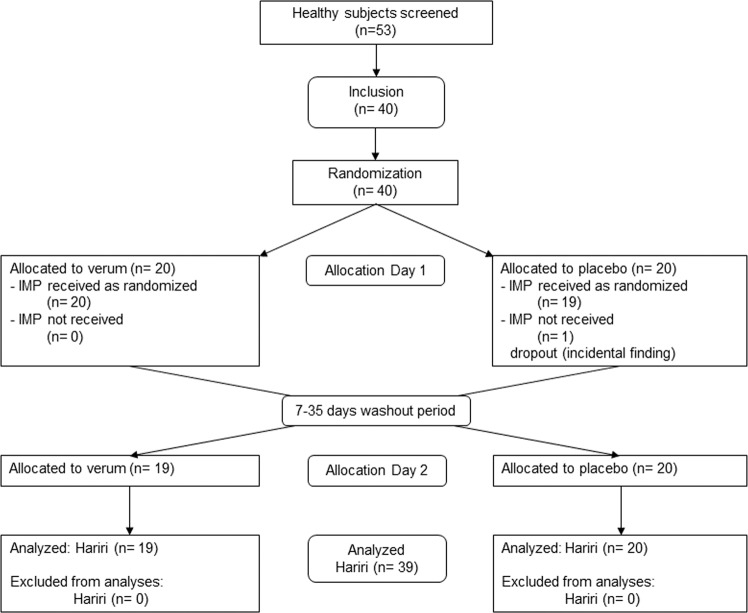
CONSORT flow diagram showing number of participants through each stage of the randomized cross-over trial.

All participants were screened 7 to 21 days before randomization and filled in questionnaires at home 3 to 7 days before the randomization visit (Day 1), which was at the same time the day of first study treatment and assessments. The second treatment (after cross-over) was administered 7 to 35 days after the first treatment on Day 2. Measurements were done on the same day. The flow of participants through each stage of the trial according to the CONSORT diagram^48^ is depicted in Fig. 7. There was one dropout due to an incidental cerebral finding at the first MR measurement. The participant did not receive the IMP.

### Intervention

The tested drug was Neurexan^®^ (Nx4) manufactured by Heel GmbH (Nx4 tablets: lot No. 71417, manufacturing date June 12^th^, 2015; placebo tablets: lot No. 71488, manufacturing date June 16^th^, 2015). Nx4 is a Medicinal Product sold over the counter (OTC) in tablet form and manufactured according to the German Regulation and international GMP standards. Ingredients of Nx4 are Passiflora incarnata (purple passionflower) D2 0.6 mg, Avena sativa (common oats) D2 0.6 mg, Coffea arabica (coffee) D12 0.6mg and Zincum isovalerianicum (valerianate of zinc) D4 0.6 mg. Additional information regarding these ingredients may be seen in more detail in Supplementary Table S1. Excipients include lactose monohydrate and magnesium stearate. Placebo tablets were composed of lactose monohydrate and magnesium stearate. Twenty participants took three tablets of Nx4 on the first measurement day (Day 1) and, after a washout period (7–35 days), received three tablets of placebo on the second measurement day (Day 2). Due to the cross-over design, the other 19 participants received placebo and Nx4 in the opposite order. The oral intake of three tablets took place 40 to 60 minutes before the second MRI scan (post-treatment scan).

### Primary outcomes

Primary outcomes were defined in a hierarchical order beforehand.

- Primary Outcome 1: Effect of drug, driven by significantly reduced amygdala activations in the contrast negative faces versus forms in verum compared to placebo conditions.
- Primary Outcome 2: Interaction of time and drug, driven by significantly greater reductions of amygdala FCD in verum compared to placebo conditions.
- Primary Outcome 3: Interaction of time and drug, driven by significantly greater changes (smaller and greater, two-sided effects because of inclusion of top-down and bottom-up processes in different regions) of amygdala-seeded connectivities in verum compared to placebo conditions.
- Primary Outcome 4: Interaction of time and drug, driven by significantly greater reductions of local resting-state activity of amygdala amplitude of low-frequency fluctuations (ALFF) in verum compared to placebo conditions.
- Primary Outcome 5: Influence of drug on directed connectivity in an effective connectivity model (Dynamic Causal Modeling [DCM]) from ventral prefrontal cortex (vPFC) towards amygdala, driven by positive effects of verum on directed negative effective (top-down) connectivity from vPFC to amygdala.
- Primary Outcome 6: Effect of drug, driven by significantly reduced stress network activation in terms of smaller activations in ACC, medio-orbitofrontal cortex, hippocampus, amygdala, and hypothalamus in the contrast (hard versus easy) in verum compared to placebo conditions.

Multiplicity due to multiple primary hypotheses was controlled by means of the principle of a priori ordered hypotheses. If the first test shows statistical significance, the second hypothesis can be tested in a confirmatory manner (with the same alpha as the first test). The same principle applies to subsequent hypotheses, as long as all preceding hypotheses show statistical significance. For the current manuscript, we focus on Primary Outcome 1. The results of Primary Outcomes 2–6 will be reported separately.

### Sample size

In this study, planned as a two-stage cross-over design with assumed correlation of 0.5 between the two periods, a two-sided 5% significance level, a power of 80%, and an effect size of Mann-Whitney statistic (MW) = 0.64 (comparable to Cohen’s d = 0.5; Colditz 1988), 2 × 17 participants were enrolled to detect effects of Nx4 in comparison to placebo. With this total sample size of 34 participants, a “medium-sized” group difference (MW = 0.64) can be detected with a power of 80%. To compensate usual ambiguities (e.g. dropouts) the final sample size was increased to 2 × 20 participants.

### Randomization

The randomization codes were generated within IDV Gauting by a statistician not involved in the study. The randomization codes and the complete generation procedure were filed at a secure place by IDV Gauting until the study database was closed. Pharmaceutical Development (Supply Chain) of Heel GmbH received pack lists with medication numbers from a statistician not involved in the study, for the purpose of manufacturing the drug kits according to the randomization list. Participants were randomly allocated to Nx4 or placebo in a 1:1 randomization. A corresponding random code list was prepared, using the random permuted block scheme with fixed block size. Participants were enrolled based on the randomization number recorded on the study medication. The next participant eligible for randomization was allocated to the lowest available randomization number at the site. The sealed random code list and the sets of sealed envelopes were prepared using the validated program RANCODE (Ver. 3.6) in a validated working environment at idv Data Analysis and Study Planning, Krailling, Germany.

### Blinding

All study personnel as well as the participants were blind to the study treatment during the study. Verum and placebo were identical in terms of taste, size, color and labeling; both contained lactose monohydrate and magnesium stearate. The investigator kept the treatment code envelopes throughout the course of the study and was not authorized to break the code without a valid reason (e.g., in case of emergency). Details of the unblinding procedure were explained in the Safety Management Plan for this study. There was no emergency unblinding in this study.

### EEG data acquisition

All EEG data was acquired using Brain Products Easy Cap with 64-channels. One channel placed on the back was used for electrocardiogram (ECG) detection. FCz was used as reference electrode and CPz as ground electrode. The sampling rate was 2500 Hz with 400 μS sampling interval. The data was recorded using BrainVision Recorder Professional V.1.20.0801. EEG data was also acquired during MRI scans to monitor the subjects’ vigilance especially during the resting-state runs. Inside the MRI scanner a large number of artefacts in the EEG is observed due to the gradients as well as movements. Using an established hardware-based artefact reduction employing carbon-wire loops (CWL) scanner artefacts are directly measured and removed from the EEG data, improving subsequent EEG data analysis^49^. We did not analyze the EEG data for this manuscript as this is not necessary for the face matching task.

### fMRI data acquisition

All MRI data were acquired on a Philips 3T scanner in Magdeburg. First, structural T1-weighted images for spatial normalization were measured using a turbo field echo (TFE) sequence with the following parameters: 274 sagittal slices covering the whole brain, flip angle = 8°, 256 × 256 matrix, voxel size 0.7 × 0.7 × 0.7 mm^3^. The functional MRI data acquired during the task were T2*-weighted echo-planar images (EPIs) consisting of 244 volumes for the Hariri task. The following scanner settings were used: 34 axial slices covering the whole brain, repetition time (TR) = 2,000 ms, echo time (TE) = 30 ms, flip angle = 90°, 96 × 94 matrix, field of view = 240 × 240 mm^2^, voxel size = 2.5 × 2.5 × 3 mm^3^. For the three resting-state sessions, 355 volumes of T2*-weighted EPIs were acquired for each session with the same parameters.

### fMRI data preprocessing

Preprocessing of fMRI data during task was done using Matlab 2016a (The Mathworks Inc., Natick, MA, USA) and SPM12 (Statistical parametric mapping software, SPM; Wellcome Department of Imaging Neuroscience, London, UK). Data were corrected for different slice timing within one volume and for correction of head motion all images were then realigned to the first image of the task. During this step, six head motion parameters (translation: x, y, z; and rotation: pitch, roll, yaw) were extracted. Co-registration of functional images to the anatomical T1 image was done to shift the images into the same space before spatial normalizing to MNI space. Segmentation of T1 images revealed estimated deformation fields, which were used on all functional images for normalization. Functional images were resampled with a resolution of 3 × 3 × 3 mm^3^. The smoothing of the data was done with a Full-Width-Half-Maximum (FWHM) kernel of 8 × 8 × 8 mm^3^.

### Statistical analyses

#### Psychometric measures

We first calculated the differences of the subjective nervousness measured by VAS between the time point directly after the ScanSTRESS task and directly before the ScanSTRESS task (VAS5 - VAS4) for the drug condition and the placebo condition separately. We then performed a paired t-test in SPSS (IBM Corp. Released 2012. IBM SPSS Statistics for Windows, Version 21.0. Armonk, NY: IBM Corp.).

#### Primary Outcome 1 (Hariri Task fMRI)

Our analyses focused on answering three questions:

1. Ascertaining reproduction of previous accounts of amygdala activation by our experiment. This was done on a whole-brain level using a second level one sample t-test in SPM on the first level general linear models (GLM) in the placebo condition. In general, a first level GLM was modeled assuming a block design for the Hariri task with identical onset times of the different blocks for each participant. The task was structured into three conditions of interest: positive faces, negative faces, and forms. Three separate regressors were constructed accordingly for the three conditions of interest, convolved with a canonical hemodynamic response function provided by SPM12. The six head motion parameters, obtained during preprocessing, were used as additional regressors of nuisance. For each participant, the contrast of interest, [negative faces - forms], was calculated and these resulting contrast images were then used for all further analyses in the second level. In the second-level analysis for a main effect of task, a one-sample t-test on whole-brain level for the placebo condition only was performed. The sequence of study medication was added as a covariate of no interest. Significance was assessed after correcting for multiple comparisons on peak-level, for an FWE-corrected p < 0.05.
2. The Primary Outcome 1 of reduced amygdala responses under Nx4 following negative faces was performed on a prespecified ROI basis, extracting the beta values from the first level contrast negative faces - forms (see 1.) for the left amygdala, anatomically defined by the AAL coordinates, using Marsbar^50^. A paired t-test analysis of beta values was performed using SPSS (IBM Corp. Released 2012. IBM SPSS Statistics for Windows, Version 21.0. Armonk, NY: IBM Corp.). Additionally, the treatment order of drug or placebo intake across the two experiment days was modeled into a GLM repeated measures. The sequence of study medication was added as between-subject factor.
3. To locate maxima of the drug effect on the [negative face - form] contrast, the respective first level contrasts for each participant (see 1.) were taken to a second level analysis of all voxels comprised by the amygdala mask. A voxelwise paired t-test, comparing verum and placebo conditions, was controlled for multiple comparison across all voxels via a search volume (small volume) correction implemented in SPM. We checked for the specific height threshold corrected for the number of comparisons within the ROI and then applied the specific height threshold of T = 2.952 for the SVC analysis. This correction applies a peak-level FWE correction^51^. The sequence of study medication was added as a covariate of no interest to the respective second level model. Note that this analysis does not affect the derivation of primary significance on the primary outcome, which is rather provided by the direct ROI analysis.

To follow a prespecified order of results, individual results for the respective primary outcomes were specified for a left sided effect in the amygdala. This reflected prior reports on a left lateralization of effects of stress or negative emotion or their within or between subject variability^15,52,53^. All primary outcomes of this study, also those not reported here, were accordingly calculated for the left amygdala. Findings outside left amygdala ROI thus are denoted exploratory.

#### Secondary outcome (correlation with psychometric measures)

For the secondary analysis, we correlated the Nx4 efficacy on the Hariri task with the Nx4 efficacy on the VAS. For Nx4 efficacy on the Hariri task our contrast of interest was [negative faces - forms]. We estimated the second level contrast by the formula: (Nx4: negative faces - forms) - (placebo: negative faces - forms), using the image calculator of SPM12 (7219)^54^. In particular, we took the regressors for the stimulus blocks negative faces and forms from the first level and calculated (negative faces under Nx4 - forms under Nx4) - (negative faces under placebo - forms under placebo) for the Hariri task. By subtracting both control conditions, the form condition and the placebo condition, we receive the sole effect of Nx4. We call it efficacy since the effect of Nx4 is greater when the difference value is higher. The Nx4 efficacy on the VAS for nervousness was added as a covariate of interest in the second level model for this analysis. Analyses focused on the difference: VAS before ScanSTRESS - VAS after ScanSTRESS (VAS4 - VAS5). In line with this, Nx4 efficacy on VAS was calculated as (VAS4 under Nx4 - VAS5 under Nx4) - (VAS4 under placebo - VAS5 under placebo). This analysis was performed on a whole-brain level. Due to the fact that the analysis was exploratory and that the medication might have modulated activity in a more broadly distributed set of cortical regions a cluster-based inference approach was taken. All voxels surpassing an initial height threshold of p < 0.001^55^ were allowed to enter a cluster with a threshold of cluster-level FWE-corrected p < 0.05.

#### Exploratory results

In order to get a more comprehensive insight into the effect of Nx4 during emotional face processing, we assessed the whole-brain results (a) of the exploratory second level contrast [negative faces - forms], contrasting placebo and Nx4. Further small volume corrected results compared the contrasts [all faces - forms] and [positive faces - forms] between Nx4 and placebo for the left amygdala ROI (b). We used the first level regressors for negative faces, positive faces and forms described in the first paragraph of Primary Outcome 1 (Hariri task fMRI) for these analyses.

- For the exploratory whole-brain analysis we used the first level contrast [negative faces - forms]. In the second-level analyses, main effects of drug were tested with a paired t-test on whole-brain level for Nx4 versus placebo condition. The sequence of study medication was added as a covariate of no interest. A cluster-based approach was applied due to the reason stated above. All voxels surpassing an initial height threshold of p < 0.001 were allowed to enter a cluster with a threshold of cluster-level FWE-corrected p < 0.05.
- To test the drug effect within the amygdala during the blocks of faces, we created the contrast [all faces - forms] in the first level analysis. The respective first level contrasts for each participant were taken to a second level analysis of all voxels comprised by the amygdala mask. A voxelwise paired t-test, comparing verum and placebo conditions, was controlled for multiple comparison across all voxels via a search volume (small volume) correction implemented in SPM. Small volume correction applied a peak-level FWE-correction, calculated across the voxels within the small volume^51^. Results were considered for SVC peak-level FWE-corrected p < 0.05 as in Primary Outcome 1.3 and, for exploration, also for lower levels of uncorrected voxel peak-levels. The sequence of study medication was added as a covariate of no interest to the respective second level model. To test the drug effect in the amygdala during the blocks of positive faces, we created the contrast [positive faces - forms] and performed the same analysis described for the contrast [faces - forms].

Figures of significant clusters were created with MRIcron (http://www.mricro.com/mricron), using the template named “ch2better.nii.gz”.

## Supplementary information

Supplementary Information.
