# Supplementary material for: fMRI Revealed Reduced Amygdala Activation after Nx4 in Mildly to Moderately Stressed Healthy Volunteers in a Randomized, Placebo-Controlled, Cross-Over Trial
Source: Sci Rep. 2020 Mar 2;10:3802. doi: 10.1038/s41598-020-60392-w (PMC7052227; doi:10.1038/s41598-020-60392-w)
Supplement: Supplementary file 1 — Supplementary Information. [file 41598_2020_60392_MOESM1_ESM.pdf]

Supplementary Material for

fMRI Revealed Reduced Amygdala Activation after Nx4 in Mildly to Moderately Stressed Healthy Volunteers in a Randomized, Placebo-Controlled, Cross-Over Trial

Luisa Herrmann[1,2], Petya Vicheva[3,4], Vanessa Kasties[1], Lena Danyeli[1,3,5], Gregor R. Szycik[6], Dominik Denzel[3,4], Yan Fan[7], Johan Van der Meer[8], Johannes C. Vester[9], Herbert Eskoetter[10], Myron Schultz[11], and Martin Walter[1,2,3,4,5] *

[1]Department of Psychiatry and Psychotherapy, University of Tübingen, Tübingen, 72076, Germany
[2]Department of Psychiatry and Psychotherapy, University of Jena, Jena, 07743, Germany
[3]Clinical Affective Neuroimaging Laboratory (CANLAB), Magdeburg, 39120, Germany
[4]Medical Faculty, Otto von Guericke University of Magdeburg, Magdeburg, 39120, Germany
[5]Leibniz Institute for Neurobiology, Department of Behavioral Neurology, Magdeburg, 39118, Germany.
[6]Department of Psychiatry, Social Psychiatry and Psychotherapy, Hannover Medical School, Hanover, 30625, Germany
[7]Department of Psychiatry, Charité-CBF, Berlin, 12203, Germany
[8]QIMR Berghofer Medical Research Institute, Brisbane, 4006, Australia
[9]idv Data Analysis and Study Planning, Krailling, 82152, Germany
[10]Medical Consultant, Cologne, 50670, Germany
[11]Biologische Heilmittel Heel GmbH, Baden-Baden, 76532, Germany

*martin.walter@med.ovgu.de

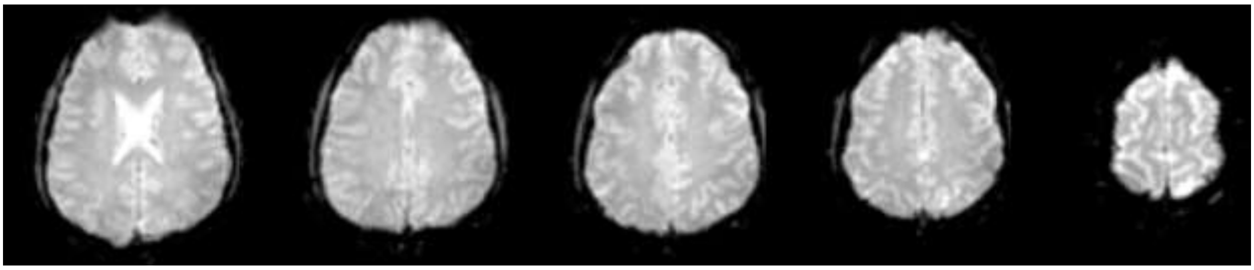

Supplementary Figure S1. Example of EPI data quality as inspected for all subjects to avoid potential degradation due to the EEG caps. As seen in the sequence of transverse sections, no signal dropouts or other artefacts are found in the data, particularly also not in the surface regions most prone to such potential effects.

Supplementary Table S1. Qualitative and quantitative composition of Neurexan®.

Active substance (homeopathic denomination)	Used plant part / Starting material<br><br>GHP method	Potency	Mass per 1 tablet (mg)	Amount per daily standard dose<br><br>(3 tablets)	Amount per maximum daily dose<br><br>(12 tablets)
**Avena sativa**<br><br>**Avena sativa L.**	Fresh, aerial parts harvested during flowering season<br>GHP method 1a<br><br>dry residue ≥ 2 %;<br><br>ǿ = ½ part pressed juice	2	0.6	1.8 mg D2<br><br>= 180 µg D1<br><br>= 36 µg mother tincture (contains 18 µg expressed juice)	7.2 mg D2<br><br>= 720 µg D1<br><br>= 144 µg mother tincture (contains 72 µg expressed juice)
**Coffea arabica (Coffea)**<br><br>**Coffea arabica L.**	Ripe, dried, unrosted seeds deprived from the exocarp<br><br>GHP method 4a;<br><br>ǿ ≥ 0.1% caffeine	12	0.6	1.8 mg D12<br><br>= 1.8 x 10^{-11} mg D1<br><br>= mother tincture with at least 1.8 x 10^{-14} mg alkaloids	7.2 mg D12<br><br>= 7.2 x 10^{-11} mg D1<br><br>= mother tincture with at least 7.2 x 10^{-14} mg alkaloids
**Passiflora incarnata**<br><br>**Passiflora incarnata L.**	Fresh aerial parts<br>GHP method 3a<br><br>dry residue ≥ 1.6 %	2	0.6	1.8 mg D2<br><br>= 180 µg D1<br><br>= 54 µg mother tincture	7.2 mg D2<br><br>= 720 µg D1<br><br>= 216 µg mother tincture
**Zincum isovalerianicum (Zincum valerianicum)**<br><br>**Zinc oxide + isovalerianic acid $Zn(C_5H_9O_2)_2$ x $2H_2O$**	Zinc isovalerianate (in German: Baldriansaures Zink)<br>GHP method 5a<br><br>D2 = 0.93 – 1.08 % substance triturations HAB 6 D1 = 9.3 – 10.8 % substance	4	0.6	1.8 mg D4<br><br>= 18 µg D2<br><br>with ca. 0.18 µg substance	7.2 mg D4<br><br>= 72 µg D2<br><br>with ca. 0.72 µg substance

Abbreviations: D = decimal potency, GHP = German Homeopathic Pharmacopoeia or Homöopathisches Arzneibuch (HAB)

Neurexan® is officially authorized by the German Authorities since 1991. It is manufactured and marketed according to the German regulation on homeopathy and contains measurable amounts of substances (Supplementary Table 1). We acknowledge that the topic of efficacy of homeopathic drugs is controversial. Two principal arguments are raised in this discussion, one - that the homeopathic drugs are diluted to extent that no single molecule is present, and another – that any beneficial effects seen in clinical trials are rather due to attentive consultation, than tested drug[1,2]. The present study prominently stands out in both of these aspects. Firstly, the test drug, Nx4, is not diluted to that level and has measurable concentrations of molecules (Supplementary Table 1). Secondly, there was no consultation in the study design, the drug was administered in a widely accepted double-blind, placebo-controlled, randomized setting to generate evidence discussed here. In this regard this study is comparable to other published clinical trials with similar design, which test drugs in fMRI paradigm[3].

References

1. Giles, J. Degrees in homeopathy slated as unscientific. *Nat.* **446**, 352 (2007).
2. Colquhoun, D. Science degrees without the science. *Nat.* **446**, 373 (2007).
3. Yue, Y. & Collaku, A. Correlation of pain reduction with fmri bold response in osteoarthritis patients treated with paracetamol: Randomized, double-blind, crossover clinical efficacy study. *Pain Medicine* **19**, 355–367 (2017).
